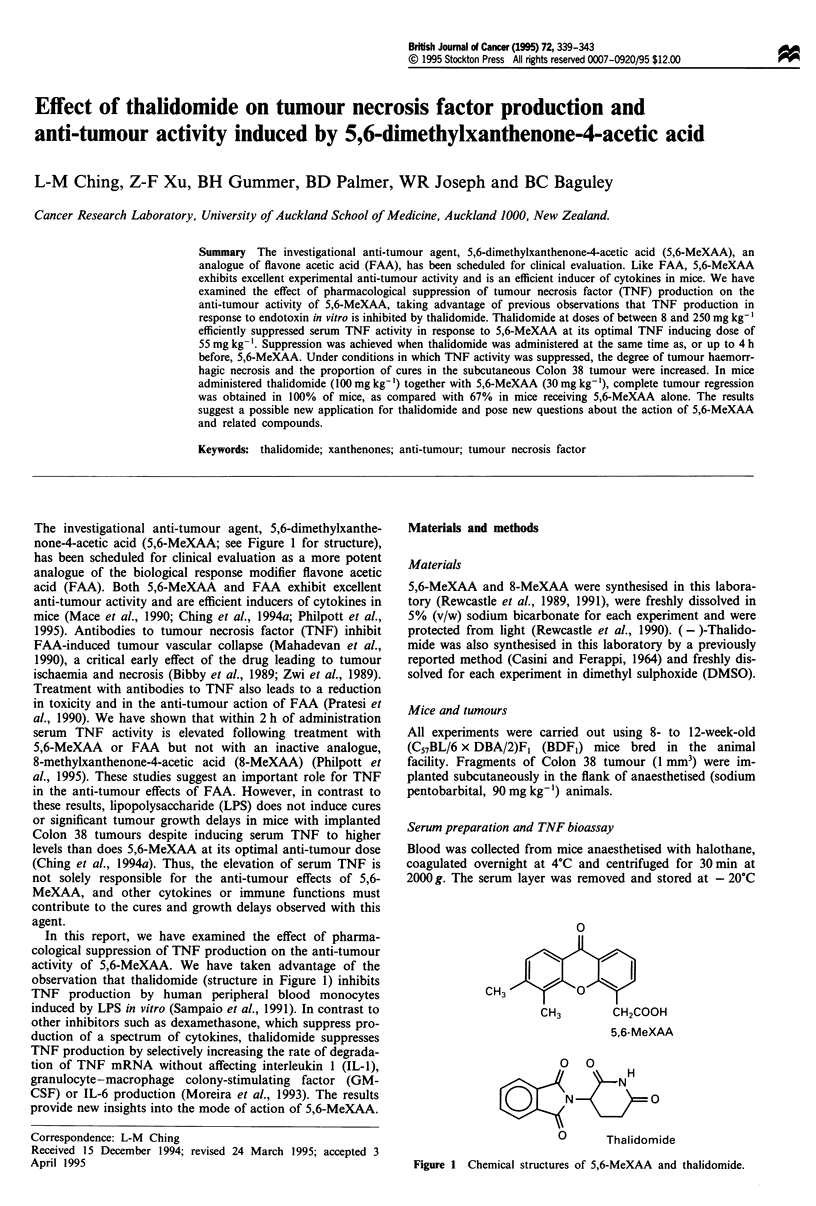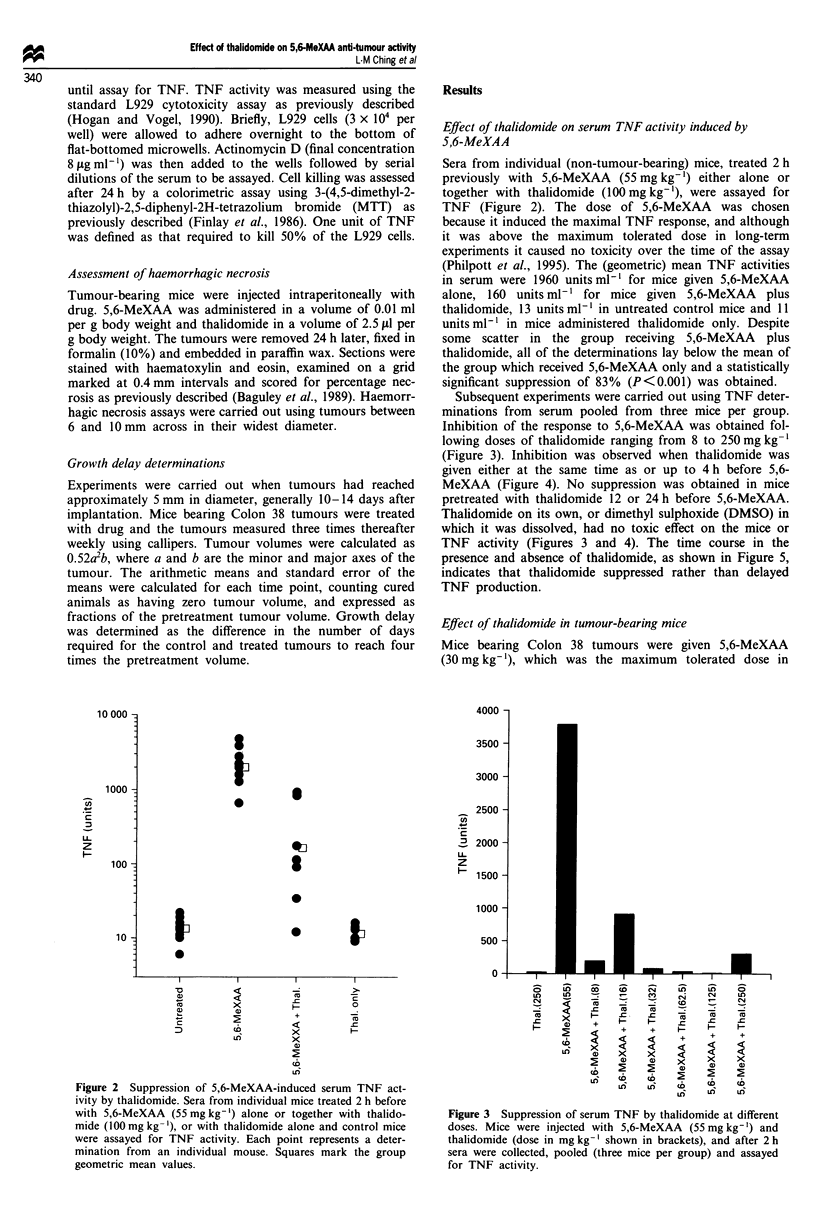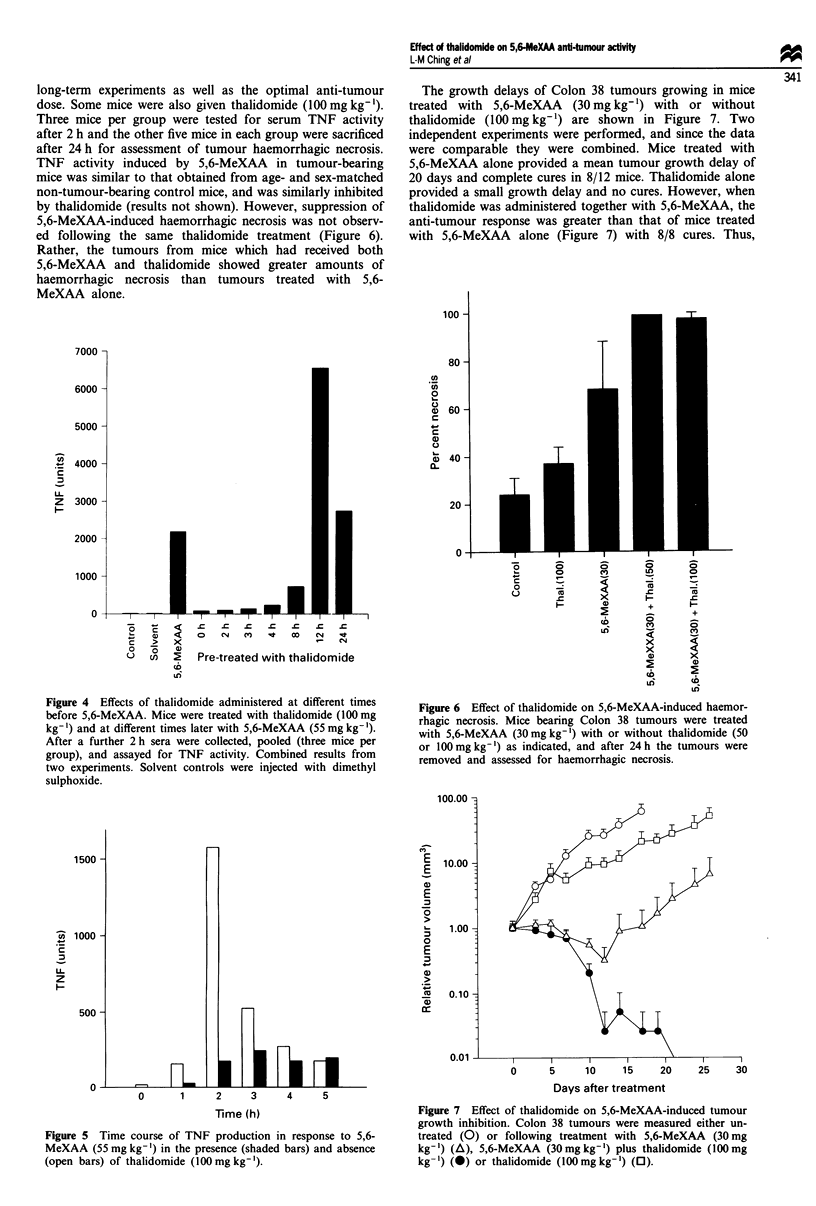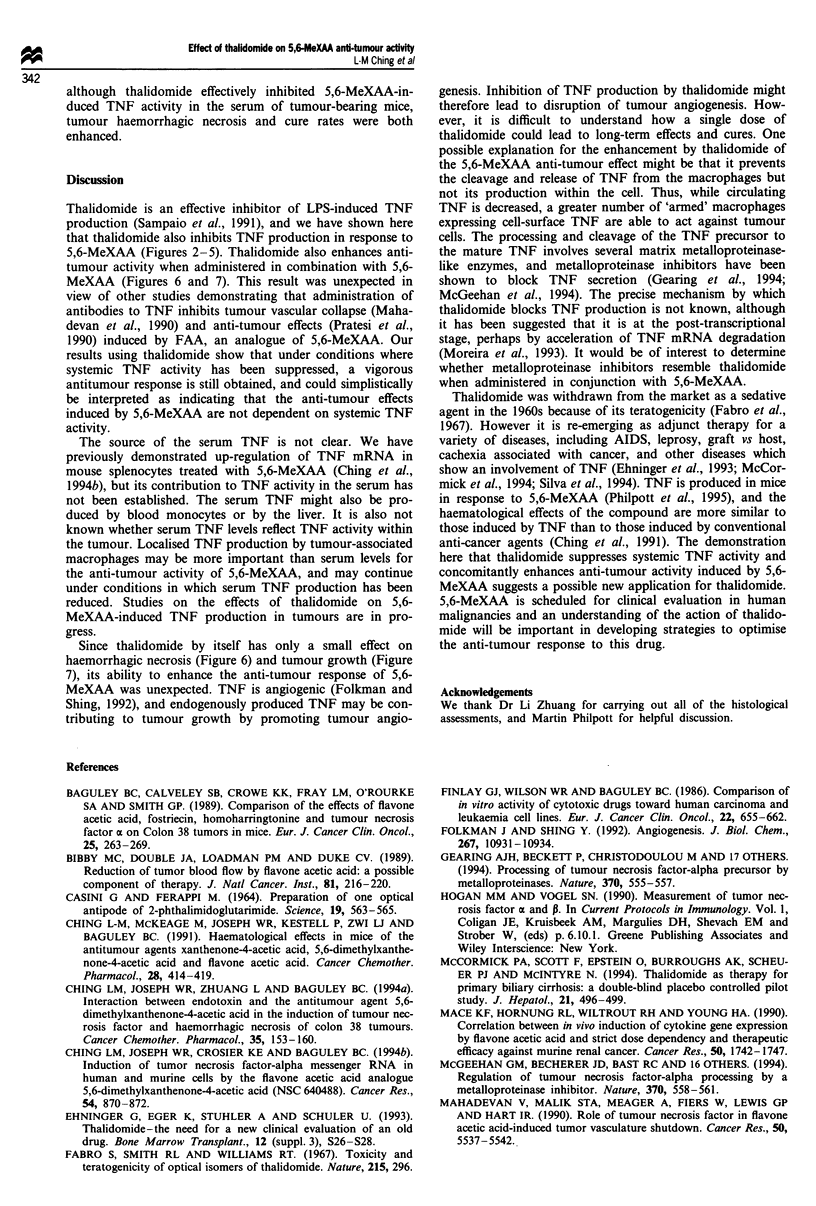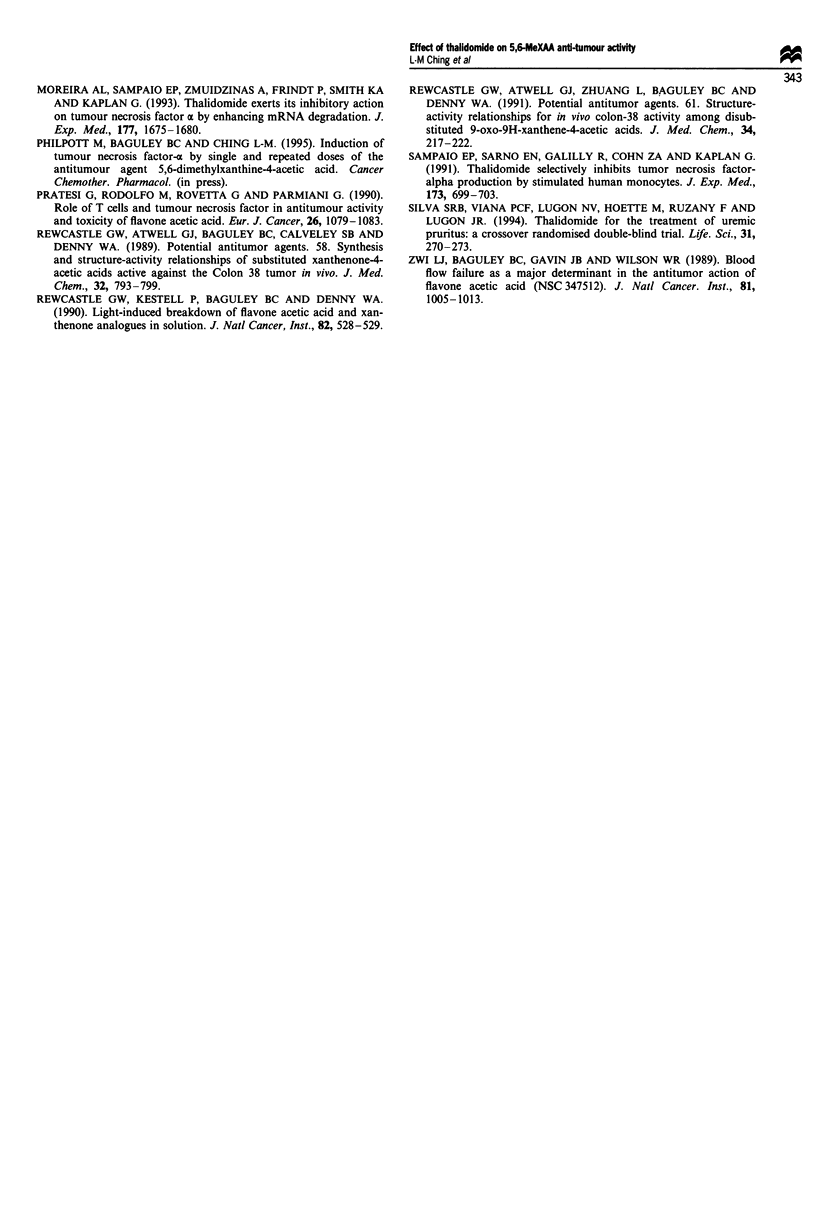# Effect of thalidomide on tumour necrosis factor production and anti-tumour activity induced by 5,6-dimethylxanthenone-4-acetic acid.

**DOI:** 10.1038/bjc.1995.335

**Published:** 1995-08

**Authors:** L. M. Ching, Z. F. Xu, B. H. Gummer, B. D. Palmer, W. R. Joseph, B. C. Baguley

**Affiliations:** Cancer Research Laboratory, University of Auckland School of Medicine, New Zealand.

## Abstract

The investigational anti-tumour agent, 5,6-dimethylxanthenone-4-acetic acid (5,6-MeXAA), an analogue of flavone acetic acid (FAA), has been scheduled for clinical evaluation. Like FAA, 5,6-MeXAA exhibits excellent experimental anti-tumour activity and is an efficient inducer of cytokines in mice. We have examined the effect of pharmacological suppression of tumour necrosis factor (TNF) production on the anti-tumour activity of 5,6-MeXAA, taking advantage of previous observations that TNF production in response to endotoxin in vitro is inhibited by thalidomide. Thalidomide at doses of between 8 and 250 mg kg-1 efficiently suppressed serum TNF activity in response to 5,6-MeXAA at its optimal TNF inducing dose of 55 mg kg-1. Suppression was achieved when thalidomide was administered at the same time as, or up to 4 h before, 5,6-MeXAA. Under conditions in which TNF activity was suppressed, the degree of tumour haemorrhagic necrosis and the proportion of cures in the subcutaneous Colon 38 tumour were increased. In mice administered thalidomide (100 mg kg-1) together with 5,6-MeXAA (30 mg kg-1), complete tumour regression was obtained in 100% of mice, as compared with 67% in mice receiving 5,6-MeXAA alone. The results suggest a possible new application for thalidomide and pose new questions about the action of 5,6-MeXAA and related compounds.


British Journal of Cancer (1995) 72, 339–343
© 1995 Stockton Press All rights reserved 0007-0920/95 $12.00

### Effect of thalidomide on tumour necrosis factor production and anti-tumour activity induced by 5,6-dimethylxanthenone-4-acetic acid

L-M Ching, Z-F Xu, BH Gummer, BD Palmer, WR Joseph and BC Baguley

*Cancer Research Laboratory, University of Auckland School of Medicine, Auckland 1000, New Zealand.*

**Summary** The investigational anti-tumour agent, 5,6-dimethylxanthenone-4-acetic acid (5,6-MeXAA), an analogue of flavone acetic acid (FAA), has been scheduled for clinical evaluation. Like FAA, 5,6-MeXAA exhibits excellent experimental anti-tumour activity and is an efficient inducer of cytokines in mice. We have examined the effect of pharmacological suppression of tumour necrosis factor (TNF) production on the anti-tumour activity of 5,6-MeXAA, taking advantage of previous observations that TNF production in response to endotoxin *in vitro* is inhibited by thalidomide. Thalidomide at doses of between 8 and 250 mg $kg^{-1}$ efficiently suppressed serum TNF activity in response to 5,6-MeXAA at its optimal TNF inducing dose of 55 mg $kg^{-1}$. Suppression was achieved when thalidomide was administered at the same time as, or up to 4 h before, 5,6-MeXAA. Under conditions in which TNF activity was suppressed, the degree of tumour haemorrhagic necrosis and the proportion of cures in the subcutaneous Colon 38 tumour were increased. In mice administered thalidomide (100 mg $kg^{-1}$) together with 5,6-MeXAA (30 mg $kg^{-1}$), complete tumour regression was obtained in 100% of mice, as compared with 67% in mice receiving 5,6-MeXAA alone. The results suggest a possible new application for thalidomide and pose new questions about the action of 5,6-MeXAA and related compounds.

**Keywords:** thalidomide; xanthenones; anti-tumour; tumour necrosis factor

The investigational anti-tumour agent, 5,6-dimethylxanthenone-4-acetic acid (5,6-MeXAA; see Figure 1 for structure), has been scheduled for clinical evaluation as a more potent analogue of the biological response modifier flavone acetic acid (FAA). Both 5,6-MeXAA and FAA exhibit excellent anti-tumour activity and are efficient inducers of cytokines in mice (Mace *et al.*, 1990; Ching *et al.*, 1994*a*; Philpott *et al.*, 1995). Antibodies to tumour necrosis factor (TNF) inhibit FAA-induced tumour vascular collapse (Mahadevan *et al.*, 1990), a critical early effect of the drug leading to tumour ischaemia and necrosis (Bibby *et al.*, 1989; Zwi *et al.*, 1989). Treatment with antibodies to TNF also leads to a reduction in toxicity and in the anti-tumour action of FAA (Pratesi *et al.*, 1990). We have shown that within 2 h of administration serum TNF activity is elevated following treatment with 5,6-MeXAA or FAA but not with an inactive analogue, 8-methylxanthenone-4-acetic acid (8-MeXAA) (Philpott *et al.*, 1995). These studies suggest an important role for TNF in the anti-tumour effects of FAA. However, in contrast to these results, lipopolysaccharide (LPS) does not induce cures or significant tumour growth delays in mice with implanted Colon 38 tumours despite inducing serum TNF to higher levels than does 5,6-MeXAA at its optimal anti-tumour dose (Ching *et al.*, 1994*a*). Thus, the elevation of serum TNF is not solely responsible for the anti-tumour effects of 5,6-MeXAA, and other cytokines or immune functions must contribute to the cures and growth delays observed with this agent.

In this report, we have examined the effect of pharmacological suppression of TNF production on the anti-tumour activity of 5,6-MeXAA. We have taken advantage of the observation that thalidomide (structure in Figure 1) inhibits TNF production by human peripheral blood monocytes induced by LPS *in vitro* (Sampaio *et al.*, 1991). In contrast to other inhibitors such as dexamethasone, which suppress production of a spectrum of cytokines, thalidomide suppresses TNF production by selectively increasing the rate of degradation of TNF mRNA without affecting interleukin 1 (IL-1), granulocyte–macrophage colony-stimulating factor (GM-CSF) or IL-6 production (Moreira *et al.*, 1993). The results provide new insights into the mode of action of 5,6-MeXAA.

Correspondence: L-M Ching
Received 15 December 1994; revised 24 March 1995; accepted 3 April 1995

### Materials and methods

### *Materials*

5,6-MeXAA and 8-MeXAA were synthesised in this laboratory (Rewcastle *et al.*, 1989, 1991), were freshly dissolved in 5% (v/w) sodium bicarbonate for each experiment and were protected from light (Rewcastle *et al.*, 1990). (–)-Thalidomide was also synthesised in this laboratory by a previously reported method (Casini and Ferappi, 1964) and freshly dissolved for each experiment in dimethyl sulphoxide (DMSO).

### *Mice and tumours*

All experiments were carried out using 8- to 12-week-old ($C_{57}BL/6 \times DBA/2)F_1$ ($BDF_1$) mice bred in the animal facility. Fragments of Colon 38 tumour (1 $mm^3$) were implanted subcutaneously in the flank of anaesthetised (sodium pentobarbital, 90 mg $kg^{-1}$) animals.

### *Serum preparation and TNF bioassay*

Blood was collected from mice anaesthetised with halothane, coagulated overnight at 4°C and centrifuged for 30 min at 2000 *g*. The serum layer was removed and stored at – 20°C

**Figure 1** Chemical structures of 5,6-MeXAA and thalidomide.

until assay for TNF. TNF activity was measured using the standard L929 cytotoxicity assay as previously described (Hogan and Vogel, 1990). Briefly, L929 cells ($3 \times 10^4$ per well) were allowed to adhere overnight to the bottom of flat-bottomed microwells. Actinomycin D (final concentration 8 $\mu g\ ml^{-1}$) was then added to the wells followed by serial dilutions of the serum to be assayed. Cell killing was assessed after 24 h by a colorimetric assay using 3-(4,5-dimethyl-2-thiazolyl)-2,5-diphenyl-2H-tetrazolium bromide (MTT) as previously described (Finlay *et al.*, 1986). One unit of TNF was defined as that required to kill 50% of the L929 cells.

### *Assessment of haemorrhagic necrosis*

Tumour-bearing mice were injected intraperitoneally with drug. 5,6-MeXAA was administered in a volume of 0.01 ml per g body weight and thalidomide in a volume of 2.5 $\mu l$ per g body weight. The tumours were removed 24 h later, fixed in formalin (10%) and embedded in paraffin wax. Sections were stained with haematoxylin and eosin, examined on a grid marked at 0.4 mm intervals and scored for percentage necrosis as previously described (Baguley *et al.*, 1989). Haemorrhagic necrosis assays were carried out using tumours between 6 and 10 mm across in their widest diameter.

### *Growth delay determinations*

Experiments were carried out when tumours had reached approximately 5 mm in diameter, generally 10–14 days after implantation. Mice bearing Colon 38 tumours were treated with drug and the tumours measured three times thereafter weekly using callipers. Tumour volumes were calculated as $0.52a^2b$, where $a$ and $b$ are the minor and major axes of the tumour. The arithmetic means and standard error of the means were calculated for each time point, counting cured animals as having zero tumour volume, and expressed as fractions of the pretreatment tumour volume. Growth delay was determined as the difference in the number of days required for the control and treated tumours to reach four times the pretreatment volume.

### Results

### *Effect of thalidomide on serum TNF activity induced by 5,6-MeXAA*

Sera from individual (non-tumour-bearing) mice, treated 2 h previously with 5,6-MeXAA (55 $mg\ kg^{-1}$) either alone or together with thalidomide (100 $mg\ kg^{-1}$), were assayed for TNF (Figure 2). The dose of 5,6-MeXAA was chosen because it induced the maximal TNF response, and although it was above the maximum tolerated dose in long-term experiments it caused no toxicity over the time of the assay (Philpott *et al.*, 1995). The (geometric) mean TNF activities in serum were 1960 units $ml^{-1}$ for mice given 5,6-MeXAA alone, 160 units $ml^{-1}$ for mice given 5,6-MeXAA plus thalidomide, 13 units $ml^{-1}$ in untreated control mice and 11 units $ml^{-1}$ in mice administered thalidomide only. Despite some scatter in the group receiving 5,6-MeXAA plus thalidomide, all of the determinations lay below the mean of the group which received 5,6-MeXAA only and a statistically significant suppression of 83% ($P < 0.001$) was obtained.

Subsequent experiments were carried out using TNF determinations from serum pooled from three mice per group. Inhibition of the response to 5,6-MeXAA was obtained following doses of thalidomide ranging from 8 to 250 $mg\ kg^{-1}$ (Figure 3). Inhibition was observed when thalidomide was given either at the same time as or up to 4 h before 5,6-MeXAA (Figure 4). No suppression was obtained in mice pretreated with thalidomide 12 or 24 h before 5,6-MeXAA. Thalidomide on its own, or dimethyl sulphoxide (DMSO) in which it was dissolved, had no toxic effect on the mice or TNF activity (Figures 3 and 4). The time course in the presence and absence of thalidomide, as shown in Figure 5, indicates that thalidomide suppressed rather than delayed TNF production.

### *Effect of thalidomide in tumour-bearing mice*

Mice bearing Colon 38 tumours were given 5,6-MeXAA (30 $mg\ kg^{-1}$), which was the maximum tolerated dose in

**Figure 2** Suppression of 5,6-MeXAA-induced serum TNF activity by thalidomide. Sera from individual mice treated 2 h before with 5,6-MeXAA (55 $mg\ kg^{-1}$) alone or together with thalidomide (100 $mg\ kg^{-1}$), or with thalidomide alone and control mice were assayed for TNF activity. Each point represents a determination from an individual mouse. Squares mark the group geometric mean values.

**Figure 3** Suppression of serum TNF by thalidomide at different doses. Mice were injected with 5,6-MeXAA (55 $mg\ kg^{-1}$) and thalidomide (dose in $mg\ kg^{-1}$ shown in brackets), and after 2 h sera were collected, pooled (three mice per group) and assayed for TNF activity.

long-term experiments as well as the optimal anti-tumour dose. Some mice were also given thalidomide (100 mg $kg^{-1}$). Three mice per group were tested for serum TNF activity after 2 h and the other five mice in each group were sacrificed after 24 h for assessment of tumour haemorrhagic necrosis. TNF activity induced by 5,6-MeXAA in tumour-bearing mice was similar to that obtained from age- and sex-matched non-tumour-bearing control mice, and was similarly inhibited by thalidomide (results not shown). However, suppression of 5,6-MeXAA-induced haemorrhagic necrosis was not observed following the same thalidomide treatment (Figure 6). Rather, the tumours from mice which had received both 5,6-MeXAA and thalidomide showed greater amounts of haemorrhagic necrosis than tumours treated with 5,6-MeXAA alone.

The growth delays of Colon 38 tumours growing in mice treated with 5,6-MeXAA (30 mg $kg^{-1}$) with or without thalidomide (100 mg $kg^{-1}$) are shown in Figure 7. Two independent experiments were performed, and since the data were comparable they were combined. Mice treated with 5,6-MeXAA alone provided a mean tumour growth delay of 20 days and complete cures in 8/12 mice. Thalidomide alone provided a small growth delay and no cures. However, when thalidomide was administered together with 5,6-MeXAA, the anti-tumour response was greater than that of mice treated with 5,6-MeXAA alone (Figure 7) with 8/8 cures. Thus,

**Figure 4** Effects of thalidomide administered at different times before 5,6-MeXAA. Mice were treated with thalidomide (100 mg $kg^{-1}$) and at different times later with 5,6-MeXAA (55 mg $kg^{-1}$). After a further 2 h sera were collected, pooled (three mice per group), and assayed for TNF activity. Combined results from two experiments. Solvent controls were injected with dimethyl sulphoxide.

**Figure 5** Time course of TNF production in response to 5,6-MeXAA (55 mg $kg^{-1}$) in the presence (shaded bars) and absence (open bars) of thalidomide (100 mg $kg^{-1}$).

**Figure 6** Effect of thalidomide on 5,6-MeXAA-induced haemorrhagic necrosis. Mice bearing Colon 38 tumours were treated with 5,6-MeXAA (30 mg $kg^{-1}$) with or without thalidomide (50 or 100 mg $kg^{-1}$) as indicated, and after 24 h the tumours were removed and assessed for haemorrhagic necrosis.

**Figure 7** Effect of thalidomide on 5,6-MeXAA-induced tumour growth inhibition. Colon 38 tumours were measured either untreated (O) or following treatment with 5,6-MeXAA (30 mg $kg^{-1}$) (Δ), 5,6-MeXAA (30 mg $kg^{-1}$) plus thalidomide (100 mg $kg^{-1}$) (●) or thalidomide (100 mg $kg^{-1}$) (□).

although thalidomide effectively inhibited 5,6-MeXAA-induced TNF activity in the serum of tumour-bearing mice, tumour haemorrhagic necrosis and cure rates were both enhanced.

### Discussion

Thalidomide is an effective inhibitor of LPS-induced TNF production (Sampaio *et al.*, 1991), and we have shown here that thalidomide also inhibits TNF production in response to 5,6-MeXAA (Figures 2–5). Thalidomide also enhances anti-tumour activity when administered in combination with 5,6-MeXAA (Figures 6 and 7). This result was unexpected in view of other studies demonstrating that administration of antibodies to TNF inhibits tumour vascular collapse (Mahadevan *et al.*, 1990) and anti-tumour effects (Pratesi *et al.*, 1990) induced by FAA, an analogue of 5,6-MeXAA. Our results using thalidomide show that under conditions where systemic TNF activity has been suppressed, a vigorous antitumour response is still obtained, and could simplistically be interpreted as indicating that the anti-tumour effects induced by 5,6-MeXAA are not dependent on systemic TNF activity.

The source of the serum TNF is not clear. We have previously demonstrated up-regulation of TNF mRNA in mouse splenocytes treated with 5,6-MeXAA (Ching *et al.*, 1994*b*), but its contribution to TNF activity in the serum has not been established. The serum TNF might also be produced by blood monocytes or by the liver. It is also not known whether serum TNF levels reflect TNF activity within the tumour. Localised TNF production by tumour-associated macrophages may be more important than serum levels for the anti-tumour activity of 5,6-MeXAA, and may continue under conditions in which serum TNF production has been reduced. Studies on the effects of thalidomide on 5,6-MeXAA-induced TNF production in tumours are in progress.

Since thalidomide by itself has only a small effect on haemorrhagic necrosis (Figure 6) and tumour growth (Figure 7), its ability to enhance the anti-tumour response of 5,6-MeXAA was unexpected. TNF is angiogenic (Folkman and Shing, 1992), and endogenously produced TNF may be contributing to tumour growth by promoting tumour angiogenesis. Inhibition of TNF production by thalidomide might therefore lead to disruption of tumour angiogenesis. However, it is difficult to understand how a single dose of thalidomide could lead to long-term effects and cures. One possible explanation for the enhancement by thalidomide of the 5,6-MeXAA anti-tumour effect might be that it prevents the cleavage and release of TNF from the macrophages but not its production within the cell. Thus, while circulating TNF is decreased, a greater number of 'armed' macrophages expressing cell-surface TNF are able to act against tumour cells. The processing and cleavage of the TNF precursor to the mature TNF involves several matrix metalloproteinase-like enzymes, and metalloproteinase inhibitors have been shown to block TNF secretion (Gearing *et al.*, 1994; McGeehan *et al.*, 1994). The precise mechanism by which thalidomide blocks TNF production is not known, although it has been suggested that it is at the post-transcriptional stage, perhaps by acceleration of TNF mRNA degradation (Moreira *et al.*, 1993). It would be of interest to determine whether metalloproteinase inhibitors resemble thalidomide when administered in conjunction with 5,6-MeXAA.

Thalidomide was withdrawn from the market as a sedative agent in the 1960s because of its teratogenicity (Fabro *et al.*, 1967). However it is re-emerging as adjunct therapy for a variety of diseases, including AIDS, leprosy, graft *vs* host, cachexia associated with cancer, and other diseases which show an involvement of TNF (Ehninger *et al.*, 1993; McCormick *et al.*, 1994; Silva *et al.*, 1994). TNF is produced in mice in response to 5,6-MeXAA (Philpott *et al.*, 1995), and the haematological effects of the compound are more similar to those induced by TNF than to those induced by conventional anti-cancer agents (Ching *et al.*, 1991). The demonstration here that thalidomide suppresses systemic TNF activity and concomitantly enhances anti-tumour activity induced by 5,6-MeXAA suggests a possible new application for thalidomide. 5,6-MeXAA is scheduled for clinical evaluation in human malignancies and an understanding of the action of thalidomide will be important in developing strategies to optimise the anti-tumour response to this drug.

**Acknowledgements**

We thank Dr Li Zhuang for carrying out all of the histological assessments, and Martin Philpott for helpful discussion.

### References

BAGULEY BC, CALVELEY SB, CROWE KK, FRAY LM, O'ROURKE SA AND SMITH GP. (1989). Comparison of the effects of flavone acetic acid, fostriecin, homoharringtonine and tumour necrosis factor α on Colon 38 tumors in mice. *Eur. J. Cancer Clin. Oncol.*, **25**, 263–269.

BIBBY MC, DOUBLE JA, LOADMAN PM AND DUKE CV. (1989). Reduction of tumor blood flow by flavone acetic acid: a possible component of therapy. *J. Natl Cancer. Inst.*, **81**, 216–220.

CASINI G AND FERAPPI M. (1964). Preparation of one optical antipode of 2-phthalimidoglutarimide. *Science*, **19**, 563–565.

CHING L-M, MCKEAGE M, JOSEPH WR, KESTELL P, ZWI LJ AND BAGULEY BC. (1991). Haematological effects in mice of the antitumour agents xanthenone-4-acetic acid, 5,6-dimethylxanthenone-4-acetic acid and flavone acetic acid. *Cancer Chemother. Pharmacol.*, **28**, 414–419.

CHING LM, JOSEPH WR, ZHUANG L AND BAGULEY BC. (1994*a*). Interaction between endotoxin and the antitumour agent 5,6-dimethylxanthenone-4-acetic acid in the induction of tumour necrosis factor and haemorrhagic necrosis of colon 38 tumours. *Cancer Chemother. Pharmacol.*, **35**, 153–160.

CHING LM, JOSEPH WR, CROSIER KE AND BAGULEY BC. (1994*b*). Induction of tumor necrosis factor-alpha messenger RNA in human and murine cells by the flavone acetic acid analogue 5,6-dimethylxanthenone-4-acetic acid (NSC 640488). *Cancer Res.*, **54**, 870–872.

EHNINGER G, EGER K, STUHLER A AND SCHULER U. (1993). Thalidomide–the need for a new clinical evaluation of an old drug. *Bone Marrow Transplant.*, **12** (suppl. 3), S26–S28.

FABRO S, SMITH RL AND WILLIAMS RT. (1967). Toxicity and teratogenicity of optical isomers of thalidomide. *Nature*, **215**, 296.

FINLAY GJ, WILSON WR AND BAGULEY BC. (1986). Comparison of *in vitro* activity of cytotoxic drugs toward human carcinoma and leukaemia cell lines. *Eur. J. Cancer Clin. Oncol.*, **22**, 655–662.

FOLKMAN J AND SHING Y. (1992). Angiogenesis. *J. Biol. Chem.*, **267**, 10931–10934.

GEARING AJH, BECKETT P, CHRISTODOULOU M AND 17 OTHERS. (1994). Processing of tumour necrosis factor-alpha precursor by metalloproteinases. *Nature*, **370**, 555–557.

HOGAN MM AND VOGEL SN. (1990). Measurement of tumor necrosis factor α and β. In *Current Protocols in Immunology*. Vol. 1, Coligan JE, Kruisbeek AM, Margulies DH, Shevach EM and Strober W, (eds) p. 6.10.1. Greene Publishing Associates and Wiley Interscience: New York.

MCCORMICK PA, SCOTT F, EPSTEIN O, BURROUGHS AK, SCHEUER PJ AND MCINTYRE N. (1994). Thalidomide as therapy for primary biliary cirrhosis: a double-blind placebo controlled pilot study. *J. Hepatol.*, **21**, 496–499.

MACE KF, HORNUNG RL, WILTROUT RH AND YOUNG HA. (1990). Correlation between *in vivo* induction of cytokine gene expression by flavone acetic acid and strict dose dependency and therapeutic efficacy against murine renal cancer. *Cancer Res.*, **50**, 1742–1747.

MCGEEHAN GM, BECHERER JD, BAST RC AND 16 OTHERS. (1994). Regulation of tumour necrosis factor-alpha processing by a metalloproteinase inhibitor. *Nature*, **370**, 558–561.

MAHADEVAN V, MALIK STA, MEAGER A, FIERS W, LEWIS GP AND HART IR. (1990). Role of tumour necrosis factor in flavone acetic acid-induced tumor vasculature shutdown. *Cancer Res.*, **50**, 5537–5542.

MOREIRA AL, SAMPAIO EP, ZMUIDZINAS A, FRINDT P, SMITH KA AND KAPLAN G. (1993). Thalidomide exerts its inhibitory action on tumour necrosis factor α by enhancing mRNA degradation. *J. Exp. Med.*, **177**, 1675–1680.

PHILPOTT M, BAGULEY BC AND CHING L-M. (1995). Induction of tumour necrosis factor-α by single and repeated doses of the antitumour agent 5,6-dimethylxanthine-4-acetic acid. *Cancer Chemother. Pharmacol.* (in press).

PRATESI G, RODOLFO M, ROVETTA G AND PARMIANI G. (1990). Role of T cells and tumour necrosis factor in antitumour activity and toxicity of flavone acetic acid. *Eur. J. Cancer*, **26**, 1079–1083.

REWCASTLE GW, ATWELL GJ, BAGULEY BC, CALVELEY SB AND DENNY WA. (1989). Potential antitumor agents. 58. Synthesis and structure-activity relationships of substituted xanthenone-4-acetic acids active against the Colon 38 tumor *in vivo*. *J. Med. Chem.*, **32**, 793–799.

REWCASTLE GW, KESTELL P, BAGULEY BC AND DENNY WA. (1990). Light-induced breakdown of flavone acetic acid and xanthenone analogues in solution. *J. Natl Cancer, Inst.*, **82**, 528–529.

REWCASTLE GW, ATWELL GJ, ZHUANG L, BAGULEY BC AND DENNY WA. (1991). Potential antitumor agents. 61. Structure-activity relationships for *in vivo* colon-38 activity among disubstituted 9-oxo-9H-xanthene-4-acetic acids. *J. Med. Chem.*, **34**, 217–222.

SAMPAIO EP, SARNO EN, GALILLY R, COHN ZA AND KAPLAN G. (1991). Thalidomide selectively inhibits tumor necrosis factor-alpha production by stimulated human monocytes. *J. Exp. Med.*, **173**, 699–703.

SILVA SRB, VIANA PCF, LUGON NV, HOETTE M, RUZANY F AND LUGON JR. (1994). Thalidomide for the treatment of uremic pruritus: a crossover randomised double-blind trial. *Life. Sci.*, **31**, 270–273.

ZWI LJ, BAGULEY BC, GAVIN JB AND WILSON WR (1989). Blood flow failure as a major determinant in the antitumor action of flavone acetic acid (NSC 347512). *J. Natl Cancer. Inst.*, **81**, 1005–1013.